# Identifying Adolescent Sleep Problems

**DOI:** 10.1371/journal.pone.0075301

**Published:** 2013-09-24

**Authors:** Michelle A. Short, Michael Gradisar, Jason Gill, Danny Camfferman

**Affiliations:** 1 School of Psychology, Flinders University of South Australia, Adelaide, South Australia, Australia; 2 Centre for Sleep Research, University of South Australia, Adelaide, South Australia, Australia; 3 Samson Institute, School of Health Sciences, University of South Australia, Adelaide, South Australia, Australia; UCL Institute of Child Health, University College London, United Kingdom

## Abstract

**Objectives:**

To examine the efficacy of self-report and parental report of adolescent sleep problems and compare these findings to the incidence of adolescents who fulfill clinical criteria for a sleep problem. Sleep and daytime functioning factors that predict adolescents’ self-identification of a sleep problem will also be examined.

**Method:**

308 adolescents (aged 13–17 years) from eight socioeconomically diverse South Australian high schools participated in this study. Participants completed a survey battery during class time, followed by a 7-day Sleep Diary and the Flinders Fatigue Scale completed on the final day of the study. Parents completed a Sleep, Medical, Education and Family History Survey.

**Results:**

The percentage of adolescents fulfilling one or more of the criteria for a sleep problem was inordinately high at 66%. Adolescent self-reporting a sleep problem was significantly lower than the adolescents who had one or more of the clinical criteria for a sleep problem (23.1% vs. 66.6%; *χ^2^* = 17.46, *p*<.001). Parental report of their adolescent having a sleep problem was significantly lower than adolescent self-report (14.3% vs. 21.1%, *p*<.001). Adolescents who reported unrefreshing sleep were 4.81 times more likely to report a sleep problem. For every hour that bedtime was delayed, the odds of self-reporting a sleep problem increased by 1.91 times, while each additional 10 minutes taken to fall asleep increased the odds 1.40 times.

**Conclusion:**

While many adolescents were found to have sleep patterns indicative of a sleep problem, only a third of this number self-identify having a sleep problem, while only a sixth of this number are indicated by parental report. This study highlights important features to target in future sleep education and intervention strategies for both adolescents and parents.

## Introduction

To date, adolescent sleep problems have been associated with a wide range of psychiatric problems, sleep disorders, behavioural antecedents, stimulant use and an increased risk of injury and motor vehicle accidents [1]–[6]. Despite the range of negative outcomes associated with sleep problems, little is known about the prevalence of adolescents who self-report a sleep problem, the prevalence of parents who report a sleep problem in their adolescent child and the proportion of adolescents who fulfil one or more clinical criteria used to indicate a sleep problem. The potential disparity between the incidence of sleep problems and the ability of adolescents and their parents to recognize a sleep problem, further bring into question the efficacy of self and parental report in the assessment of adolescent sleep problems.

To aid with the detection and diagnosis of adolescent sleep problems, clinical indicators of sleep dysfunction highly prevalent in this population are used [3], [7]–[10]. Between 20% to 26.8% of adolescents report sleep onset latencies of 30 minutes or more [7]–[9], [11], [12] which can indicate a heightened risk of sleep onset insomnia or delayed sleep phase disorder [12], [13]. A large number of teenagers report obtaining less than 8 hours of sleep on a school night [14]–[16] which denotes a heightened risk of chronically insufficient sleep [17]–[21]. Adolescents obtaining less than 8 hours of sleep per night have been shown to have poorer working memory performance than those obtaining more than 8 hours [22]. As with adults [23], teenagers that report wake periods of more than 30 minutes during the sleep period is suggestive of sleep maintenance problems [13], [23]. Up to 44% of teenagers have a weekend bedtime delay of 2 hours or more [6], [9], [24], [25] which can indicate a Delayed Sleep Phase Disorder [DSPD; [6], [26]–[29]. Finally, difficulty in waking in the morning is symptomatic of insufficient sleep and/or DSPD [30] with one group reporting that 63% of teens are tired upon awakening [31], and another group reporting that 61% of teens being too sleepy to get out of bed in the morning [32].

Unfortunately, few studies have either asked adolescents about whether they perceive that they have a sleep problem, or looked at these clinical indicators within a community sample. One study that did is the National Sleep Foundation’s 2006 *Sleep in America Poll* [9]. This study found that teenagers self-reported having at least one or more clinical indicators of a sleep problem with 26% taking longer than 30 minutes to fall asleep; 45% sleeping less than 8 hours per night on school nights; 17% sleeping less than 8 hours per night on weekends and 37% having a weekend bedtime delay of 2 or more hours. Despite subjective reports of clinically-defined indicators of sleep problems ranging from 17% to 45%, only 16.1% of teenagers in this study felt that they had a sleep problem. The factors that differentiated adolescents who self-reported a sleep problem from those who did not, were whether they reported sleeping less than 8 hours per night, a discrepancy between school night and weekend bedtimes of more than 2 hours, and more depressed mood [9]. Unfortunately, this study relied upon single-item self-report measures of sleep. As opposed to sleep diaries or actigraphy, these items may be more susceptible to negative response bias, which would inflate the association between self-report measures.

If adolescents potentially under-identify or under-report the presence of a sleep problem, it may be beneficial to examine parental reports. Several studies have utilized parental report to describe the sleep problems of their children (aged 2 to 13 years) [33]–[35], however, few have examined the prevalence of parent-reported sleep problems in their teenagers compared with the sleep problems reported by adolescents themselves. The National Sleep Foundation’s 2006 *Sleep in America Poll* [9] reports that the incidence of parents whose adolescent was having a sleep problem was only half that of teenage self-reported sleep problems (16% vs. 7% respectively). Further, of these, only 4% of parents and teens were in agreement about the presence of a sleep problem, which is surprisingly low. This has important ramifications for the identification and treatment of adolescent sleep problems. For example, if a parent does not perceive a sleep problem, the likelihood that a teen will have access to treatment for their sleep problem may be reduced. Conversely, parents who do perceive a sleep problem may be more likely to engage in sleep-protective behaviours. The input of parental perception of an adolescent sleep problem could determine whether parents maintain appropriate limits around adolescent bedtimes to ensure adequate sleep [36] and improvements in sleep hygiene [37]. The early identification and treatment of sleep problems is therefore important in diminishing these poor habits before adulthood and further, in reducing the risk of a sleep disorder or secondary mood disorder [38]–[40].

The present study will therefore compare adolescents who self-report a sleep problem with adolescents that meet one or more of the clinical criteria for a possible sleep problem. It is hypothesized that the proportion of adolescents who report a sleep problem will be significantly smaller than the proportion who meets one or more of the clinical indicators of a sleep problem. This study will also examine parental report of sleep problems in their adolescents compared to adolescent self-report. It is hypothesized that the proportion of parents who report a sleep problem in their teen will be significantly smaller than the proportion of adolescents who self-report a sleep problem. Finally, this study will use sleep diary data to examine the hypothesis that clinical indicators such as later bedtimes, longer sleep onset latencies, shorter sleep durations, more wake after sleep onset, longer weekend bedtime delay, unrefreshing sleep, and greater sleepiness, fatigue and depressed mood will predict adolescent self-report of a sleep problem.

## Methods

### Participants

Participants were 385 adolescents (aged 13 to 18 years) recruited from 8 high schools in South Australia. Schools were selected by ranking all mainstream high schools in the Australian Bureau of Statistics Statistical Divisions of Adelaide and Outer Adelaide according to proportion of students receiving government assistance with education costs (School Card Scheme). This ranked list was divided into 8 strata and 1 school was randomly selected from each stratum. One class in each of Years 9, 10 and 11 participated from each school. Of these participants, 308 (80%) returned a completed parent survey in addition to self-report surveys and sleep diaries. The present study reports data from the 308 participants with completed sleep diaries, self-report *and* parent surveys. These adolescents did not differ significantly from those who did not have a completed parent survey in terms of age, sex, socioeconomic status (school stratum) or self-reported sleep problem (all *p*>.05).

### Measures

The School Sleep Habits Survey (SSHS) [6] contains questions regarding demographic, sleep and lifestyle variables. Adolescent-reported sleep problems were measured using an item drawn from the SSHS, “*Do you think that you have a sleep problem?*” Adolescents could indicate “*Yes*” or “*No*” and, if yes, they were asked to indicate why, using an open-ended format. In order to assess refreshing sleep, an additional Yes/No item was added to the SSHS by the authors. This item asked “*Do you wake up feeling that your sleep has not been refreshing?*”.

Difficulty waking in the morning was measured using an item drawn from the Composite Morningness Eveningness Scale [41]. This item asks, “*Assuming normal circumstances, how easy do you find getting up in the morning? (Check one)*”. Answers included “*Not at all easy*,” “*Slightly easy*,” “*Fairly easy*,” and “*Very easy*.” Respondents indicating “*Not at all easy*” were classified by the authors as having difficulty waking in the morning.

The Sleep, Medical, Education and Family History Survey [42] was developed by Prof. Mary Carskadon and colleagues at the Sleep for Science research laboratory at Brown University and completed by parents in the present study. It contains items regarding their adolescent’s sleep, parent education and employment, and family routines. Parent reports of adolescent sleep problems were assessed using the Yes/No item, “*Do you think that your child has a sleep problem?*” If parents indicated yes, they were asked to indicate why in an open-ended format. The responses of both the parents and adolescents who indicated a sleep problem were coded by two independent assessors. Initial inter-rater agreement was 94.6%. Following consultation, the two raters were able to agree on the classifications of the remaining responses.

A 7-day sleep diary was used to collect information on adolescents’ daily bedtime, sleep onset latency, wake after sleep onset (WASO), total sleep time (TST), and wake time. Participants were instructed to fill out their sleep diary morning and night and to indicate an exact time for all variables. Participants also phoned their bedtime and wake time to the Flinders University Sleep Laboratory, morning and night, in order to ensure that sleep diaries were being filled out contemporaneously and not retrospectively. Weekend delay was calculated by subtracting adolescents’ mean school night bedtime (Sunday to Thursday nights) from their mean weekend bedtime (Friday and Saturday nights). Good correspondence has been shown between sleep diaries and polysomnography (kappa = 0.87), with high sensitivity (92.3%) and specificity (95.6%) [43].

The Centre for Epidemiological Studies Depression Scale (CES-D) [44] is a 20-item scale assessing depressed mood. Items include “*I felt sad,*” and “*I had crying spells*.” Adolescents indicated how often each item applied to them in the last week on a 4-point scale, where 0 =  rarely or none of the time (less than one day) and 3 =  most or all of the time (5 to 7 days). Total scores range from 0 to 60, with higher scores indicated more depressed mood. Adolescents were classified as having elevated depression using the cut-offs developed by Garrison and colleagues [45], being a total score of 12 or higher for boys and 22 or higher for girls. The factor structure of the CES-D has been shown to apply equally well in adults and adolescents, and has displayed longitudinal and gender invariance in a study of 2,416 adolescents and good concurrent validity [46].

The Flinders Fatigue Scale (FFS) [47] is a 7-item scale assessing the experience of fatigue over the previous two weeks. Items included “*Was fatigue a problem for you?*” and “*How severe was the fatigue you experienced?*” Responses ranged from 0 “*Not at all*” to 4 “*Extremely.*” Total scores could range from 0 to 31, with higher scores indicating greater fatigue. Previous research has reported good reliability and validity of this scale amongst adult good sleepers and adults with insomnia [43]. Poor sleepers reported significantly greater levels of fatigue than good sleepers, showing good discriminant validity.

Daytime sleepiness was measured using the Pediatric Daytime Sleepiness Scale (PDSS) [21]. This 8-item scale asks adolescents about their experience of sleepiness over the previous two weeks. Items included “*How often do you fall asleep or feel drowsy in class?*,” and “*Are you usually alert during the day?*” Responses ranged from 0 “*Never*” to 4 “*Frequently*.” Total scores could range from 0 to 32, with higher scores indicating greater daytime sleepiness. The PDSS was developed and validated on a sample of 450 children and adolescents, aged 11 to 15 years. Factor analyses of two split-half samples supported a one-factor solution which accounted for 32% of the variance. Higher scores on the PDSS were associated with poorer school achievement, less enjoyment of school, absenteeism, illness, and worse mood [19].

### Procedure

Written informed consent was obtained from school Principals, adolescents and their parent/guardian. The survey battery was administered during class time at school. Participants completed a 7-day sleep diary for the following week and a parent or guardian completed the Sleep, Medical, Education and Family History Survey. On the final day of the study, participants returned all study materials and completed the Flinders Fatigue Scale. The study had no exclusion criteria and adolescents were reimbursed for their time with an AUD$40 gift voucher.

### Ethics

The Flinders University Social and Behavioural Research Ethics Committee and the Department of Education and Children’s Services approved this study.

## Results

### Demographics

The mean (*SD*) age of participants was 15.6 years (*SD* = 0.94). The proportion of males participating in the study was 59%. In addition to standard demographic information, details were provided as to the teenagers home life and sleeping arrangements. The majority of adolescents (78.9%) resided with two parents and had their own bedroom (83.8%). Nearly 80% of fathers and 30% of mothers were engaged in fulltime paid employment. Approximately two thirds of parents were born in Australia (69.7% of mothers and 66.8% of fathers), as were 86.6% of adolescents.

### Incidence of Adolescent’s and Parent’s Reporting a Sleep Problem

Of the 308 families in the present study, 23.1% of adolescents self-reported a sleep problem, 14.3% of parents reported a sleep problem in their teen, yet 66.6% of adolescents had one or more clinical indicators of a sleep problem (see Table 1). Chi-square analyses showed that adolescents were significantly more likely to report that they had a sleep problem than their parents *(*23.1% vs. 14.3%; *χ^2^* = 42.48, *p*<.001).

**Table 1:** The proportion of adolescents with individual clinical indicators of a sleep problem (all estimate are from sleep diary reports on school nights).

Item	Percent
Total sleep less than 8 hours per night	36.0%
Sleep onset latencies of more than 30 minutes per night	15.5%
Weekend bedtime delay of 2 hours or more	21.5%
WASO of more than 30 minutes per night	4.7%
Difficulty waking in the morning	26.3%
Depressed mood	35.1%

### Comparison between Adolescent and Parental Report of a Sleep Problem

Interestingly, the parents who reported a sleep problem in their teen did not necessarily have a teen who self-reported a sleep problem. In 8.8% of cases adolescents and parents agreed that the teen did have a sleep problem. In 5.5% of cases parents thought that their teen had a sleep problem but the adolescent did not, while in 14.3% of cases adolescents thought that they had a sleep problem but their parent did not. The percentage of adolescents who self-reported a sleep problem was compared with the percentage who reported one or more clinical indicators of a sleep problem. The proportion of adolescents self-reporting a sleep problem was significantly smaller than adolescents those who reported one or more clinical indicators of a sleep problem (23.1% vs. 66.6%; *χ^2^* = 17.46, *p*<.001).

### Sleep Factors Indicating a Sleep Problem

**Figure 1:**
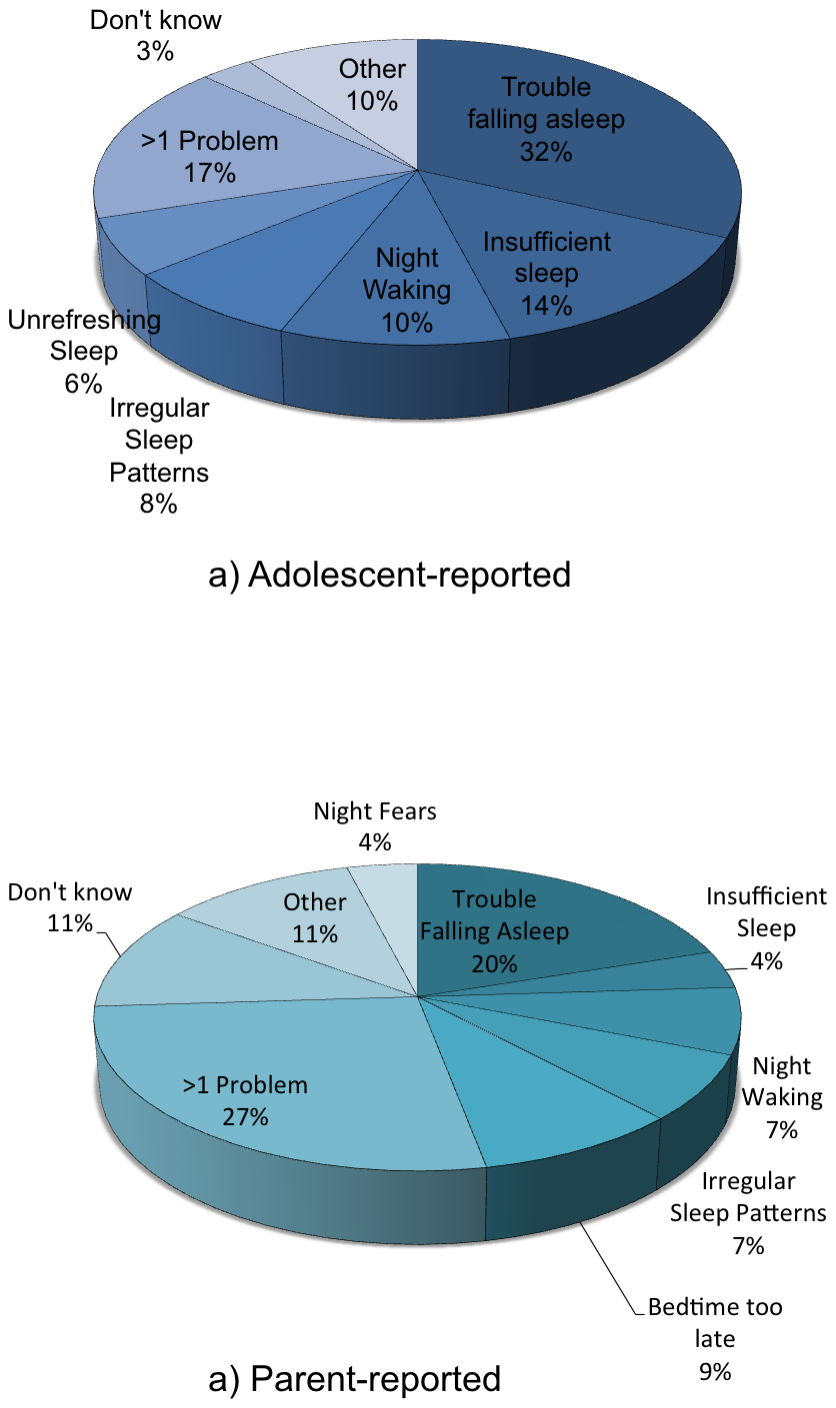
The main reasons provided by adolescents and parents for reporting that the adolescent has a sleep problem.

Adolescents and parents who had reported the incidence of an adolescent sleep problem were asked to provide their reasons (Figure 1). Difficulty initiating sleep was the most common reason given by both adolescents and their parents. Among both adolescents and parents who provided more than one reason, common responses included sleep factors (e.g., difficulty falling asleep, irregular sleep patterns and trouble waking), and daytime functioning factors (e.g., feeling tired). Parents often reported morning or daytime grumpiness as one of the reasons they thought their teen had a sleep problem, compared to none of the teens. Responses from adolescents that were included in the “Other” category included a rhythmic movement disorder, sleep apnoea, being woken with indigestion, and being woken regularly by a crying sibling. Among parents, responses in the “Other” category included grumpiness upon awakening, sleep apnea, a rhythmic movement disorder, and bedtime resistance.

Bivariate and point biserial correlations between the variables included in the logistic regression are shown in Table 2. A logistic regression analysis was used to determine which factors would predict whether or not an adolescent reported a sleep problem. Age and sex were not included in the analysis as there were no differences in these variables between groups, both *p*>.05. Sleep parameters were entered into Step 1. Daytime functioning variables were entered in Step 2. The daytime functioning variables were entered into the second step of the logistic regression to determine whether depressed mood, sleepiness or fatigue would predict sleep problems over and above the variance they share with sleep. In Step 1, the test of the model coefficient was significant, *χ^2^* = 63.85, *p*<.001, with 32.8% of the variance in sleep problem status explained by the predictor variables. In Step 2, the addition of the daytime functioning variables explained an additional 6.6% of the variance in sleep problem status, *χ^2^* = 14.89, *p*<.01. Overall, the full model was significant, *χ^2^* = 78.74, *p*<.001, and explained 39.4% of the variance in sleep problem status (Nagelkerke R^2^). This model correctly classified 84.1% of cases. The contribution of each predictor is reported in Table 3. Results showed that sleep diary reported bedtime, sleep latency, and sleep refreshment each made a significant, unique contribution. Every extra 10 minutes of trying to initiate sleep increased the odds of a teen-reported sleep problem by 1.40 times, while each one hour later that bedtime occurred increased the odds of the adolescent reporting a sleep problem by 1.91 times. Adolescents who reported experiencing sleep that was not refreshing were 4.81 times more likely to report a sleep problem.

**Table 2:** Correlations between variables entered into the logistic regression.

	Depression†	Fatigue	Sleepiness	Unrefreshing Sleep	Weekend Delay	WASO	TST	SOL	Bedtime
Sleep Problem†	.29***	.41***	.32***	.37***	−.04	.11	−.24***	.24***	.24***
Bedtime	.20**	.29***	.33***	.22***	−.24***	−.12	−.50***	−.03	
SOL	.18**	.17*	.04	.08	.07	.16*	−.18**		
TST	−.19**	−.30***	−.10	−.19**	.17**	−.01			
WASO	.02	.06	.05	.16*	.21**				
W/E Delay	−.09	−.10	.10	.00					
Unrefr. sleep†	.34***	.46***	.41***						
Sleepiness	.32***	.57***							
Fatigue	.34***								

*** *p*<.001, **p<.01, *p<.05.

† Sleep problems, depressed mood and unrefreshing sleep are dichotomous categorical variables that are scored such that a higher score indicates the presence of that variable.

**Table 3:** Predictors of an adolescent self-reported sleep problem.

	B	S.E.	Wald	*p*	O.R.	95% C.I.
**Step 1**						
Bedtime	.65	.25	6.61	.01*	1.91	1.16–3.13
SOL	.04	.01	12.53	<.001*	1.04	1.02–1.06
TST	−.004	.004	1.32	.25	1.00	.99–1.00
WASO	.02	.02	1.69	.19	1.02	.99–1.05
Weekend Delay	.13	.14	.85	.36	1.14	.86 – 1.51
Unrefresh. sleep	1.57	.38	16.91	<.001*	4.81	2.27–10.15
**Step 2**						
Sleepiness	.03	.05	.42	.52	1.03	.94–1.13
Fatigue	.08	.04	3.85	.05	1.09	1.00–1.18
Depression (Y/N)	.76	.40	3.72	.05	2.14	.99–4.64

* *p*<.05.

## Discussion

The present study found that significantly more adolescents report having clinical indicators of having a sleep problem than adolescents that self-reported having a sleep problem, and further, that the proportion of parents who report an adolescent sleep problem was significantly smaller than adolescent report. Furthermore, poor concordance between adolescent and parent report of a sleep problem brings into question the efficacy of these measures. These findings support the incongruity between adolescents self-report and parental report of their adolescent having a sleep problem found in the 2006 Sleep in America Poll [9]. In the present sample there were a larger number of both adolescents and parents who reported a sleep problem, compared to those in the 2006 U.S. poll (adolescents 21% vs. 16% and parents 14% vs. 7%, respectively). However, it is unclear whether this difference may reflect cultural differences, or whether people are better aware of factors constituting good sleep.

There are a number of reasons why parents may under-report sleep problems in their adolescent. Unlike younger children, adolescents may be more likely to attempt self-management of sleep problems such as trouble falling asleep or night-time waking. Thus, parents may be unaware of the sleep patterns of their teenagers beyond their time in bed. This study found that parents were more reliant on daytime indicators of sleep problems, such as difficulty waking their teen in the morning and daytime grumpiness and sleepiness (see Figure 1).

Parents may also be unaware of what constitutes “good” sleep in adolescents, perhaps expecting that they will have the same sleep duration as adults to maintain optimal functioning, or that irregular sleep patterns and sleeping in are as common in the adolescent population as to be considered ‘normal’. This has important ramifications for adolescent sleep and well-being. Parental involvement in maintaining healthy sleep schedules has been shown to benefit adolescent sleep and daytime functioning [36], [48]. However, parents are unlikely to modify family, homework, or sleep schedules if they do not believe that there is a problem with their teens’ sleep. Further, if many parents are unaware that their adolescent has a sleep problem, there is a risk these adolescents will not gain access to treatment.

The present study found that a significantly higher proportion of adolescents had one or more clinical indicators of a sleep problem compared to the percentage who self-reported a sleep problem. The most common clinical indicators were sleeping less than 8 hours per night, difficulty waking in the morning and a weekend bedtime delay of more than 2 hours. In contrast, the most common reasons given by adolescents for having a sleep problem were difficulty falling asleep, insufficient sleep and night-time waking. Thus, the salience of particular sleep problems may differ between clinical and adolescent report. Other factors, such as perceived social norms and controllability may play a role in these differences. Adolescents may make comparisons against their peers to determine whether their sleep is problematic or “normal”. Given the high prevalence of poor sleep in this age group, this may contribute to teens under-identifying sleep problems. In addition, the likelihood that an adolescent will classify their sleep as problematic may be heightened if the problem is perceived as being beyond their control. This could partially explain why teens are more likely to report trouble falling asleep and nighttime waking as prominent reasons for thinking that they have a sleep problem, even though insufficient sleep and trouble waking in the morning are more prevalent. Teens may feel that they could obtain more sleep if they had the opportunity or if they adjusted their lifestyle, thus feeling more alert in the morning, whereas falling asleep quickly and maintaining sleep is perceived as beyond their control. While this is speculative, this reveals a potentially important area for future research.

School-night bedtimes and sleep onset latencies, together with sleep refreshment, were significant predictors of sleep problem status. Adolescents who reported unrefreshing sleep were significantly more likely to report a sleep problem than those who did not. Later adolescent bed times and longer time taken to fall asleep were also defining features. Longer sleep latencies and unrefreshing sleep are consistent with adolescents’ reasons for indicating that they had a sleep problem. More surprising, it was later bedtimes, and not total sleep that significantly predicted sleep problem status, even though late bedtimes were not a reason self-reported by adolescents as a reason for thinking that they have a sleep problem. Finally, daytime functioning variables were not significant predictors of sleep problem status over and above the variance that they share with sleep variables.

### Limitations of the Present Study

The limitation of measuring difficulty waking in the morning with one binary item must be acknowledged. Due to the cross-sectional nature of this study, causation cannot be determined (in terms of what predicts a sleep problem) and other explanations for these findings must be considered. One other potential explanation for these findings is that the relationships between self-reported sleep problems and self-reported sleep patterns and daytime functioning are inflated due to a negative response bias. Adolescents who self-report a sleep problem may report all aspects of their sleep and functioning more negatively. While sleep diary reports are arguably less susceptible to this response bias than survey reports of sleep, this remains an important aspect to consider.

### Concluding Remarks

The present study adds to our understanding of the factors that determine why adolescents self-report a sleep problem, what predicts this self-identification, and the significant discrepancies between adolescent-report, parent-report and clinical indicators. These discrepancies are of concern because they have important implications for the well-being of adolescents. These results further highlight the need for on-going sleep education to build upon adolescents’ knowledge of what constitutes healthy adolescent sleep [49], and to also educate their parents on what comprises of healthy teen sleep and possible warning signs of a potential sleep problem.

## Acknowledgments

The authors wish to that the school principals, teachers, adolescents and parents/caregivers for their generous contributions to this study. We also wish to thank Dr Hayley Dohnt, Anna Johnston, Michele Finlay and Laura Jarema for their assistance with data collection.
